# Supplementary material for: Diagnostic Accuracy of Serum Hyaluronan for Detecting HCV Infection and Liver Fibrosis in Asymptomatic Blood Donors
Source: Molecules. 2021 Jun 25;26(13):3892. doi: 10.3390/molecules26133892 (PMC8270308; doi:10.3390/molecules26133892)
Supplement: Supplementary file 1 [file molecules-26-03892-s001.zip › molecules-1223294-SI.pdf]

molecules

Supplementary Material

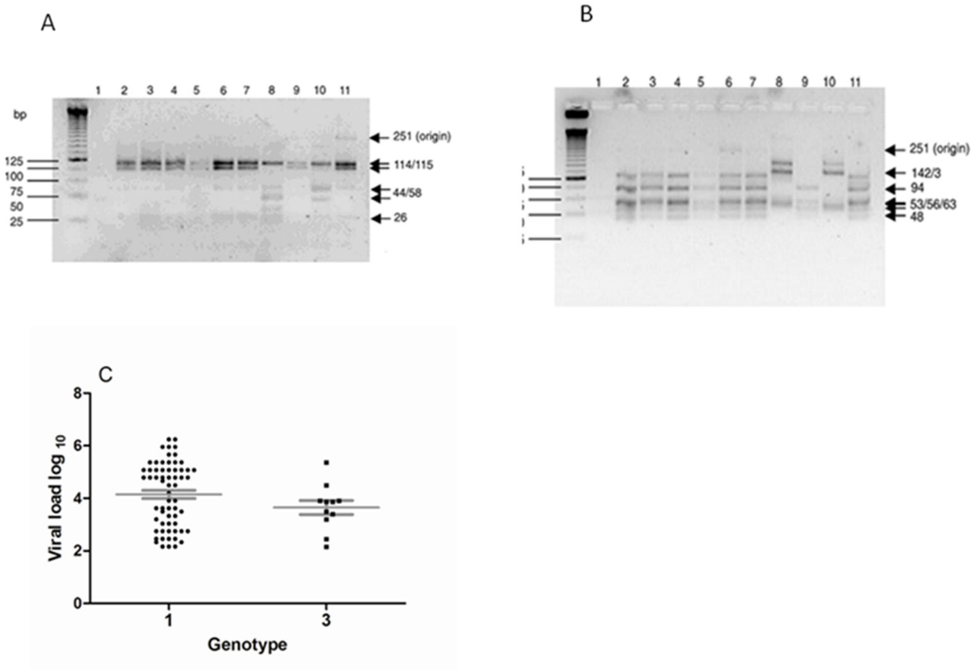

Figure 1S. HCV genotyping of blood donors. (1S A) Eletrophoresis results of enzymes RsaI and HaeIII digestion; (1S B) Eletrophoresis results of enzymes HinfI and MvaI digestion; Line 1: negative control; lines 2-7, 9 and 11: genotype 1 samples; line 8 and 10: genotype 3 samples; Used 25 bp DNA ladder. Genotypes were deduced from the combination of band patterns generated in two reactions of restriction. (1S C) HCV blood donors viral load in function of genotypes 1 (N=82) and 3 (N=13). Mean ± SD; unpaired t- test, P > 0.05, not significant.
